# Persistent Spike-specific T cell immunity despite antibody reduction after 3 months from SARS-CoV-2 BNT162b2-mRNA vaccine

**DOI:** 10.1038/s41598-022-07741-z

**Published:** 2022-04-23

**Authors:** Chiara Agrati, Concetta Castilletti, Delia Goletti, Alessandra Sacchi, Veronica Bordoni, Davide Mariotti, Stefania Notari, Giulia Matusali, Silvia Meschi, Linda Petrone, Alessandra Aiello, Saeid Najafi Fard, Chiara Farroni, Francesca Colavita, Daniele Lapa, Sara Leone, Alessandro Agresta, Maria Capobianchi, Giuseppe Ippolito, Francesco Vaia, Vincenzo Puro, INMI COVID-19 Vaccine Study Group, Eleonora Cimini, Eleonora Tartaglia, Rita Casetti, Germana Grassi, Flavia Cristofanelli, Andrea Capri, Annapaola Santoro, Nicoletta Orchi, Aurora Bettini, Massimo Francalancia, Eliana Specchiarello

**Affiliations:** INMI L.Spallanzani–IRCCS, Via Portuense 292, 00149 Rome, Italy

**Keywords:** Immunology, RNA vaccines

## Abstract

Vaccine is the main public health measure to reduce SARS-CoV-2 transmission and hospitalization, and a massive scientific effort worldwide resulted in the rapid development of effective vaccines. This work aimed to define the dynamics and persistence of humoral and cell-mediated immune response in Health Care Workers who received a two-dose BNT162b2-mRNA vaccination. Serological response was evaluated by quantifying anti-RBD and neutralizing antibodies while cell-mediated response was performed by a whole blood test quantifying Th1 cytokines (IFN-γ, TNF-α, IL-2) produced in response to Spike peptides. BNT162b2-mRNA vaccine induced both humoral and cell-mediated immune response against Spike in all HCW early after the second dose. After 12 weeks from vaccination, the titer of anti-RBD antibodies as well as their neutralization function decreased while the Spike-specific T-cells persisted at the same level as soon after vaccine boost. Of note, a correlation between cellular and humoral response persevered, suggesting the persistence of a coordinated immune response. The long lasting cell-mediated immune response after 3 months from vaccination highlight its importance in the maintaining of specific immunity able to expand again to fight eventual new antigen encountering.

## Introduction

Large-scale vaccination is the single most effective public health measure for mitigation of the coronavirus disease (COVID-19) pandemic and a global effort to develop and distribute an effective vaccine produced several effective options.

Several data are currently available about the effectiveness of BNT162b2 (two-dose vaccination regimen administered 21 days apart) in inducing a strong antibody and cell-mediated immune response in healthy individuals with a 95% efficacy in naïve individuals^1–3^. Moreover, the coordinate induction of both humoral- and cell-mediated arms has been clearly demonstrated more effective in fighting SARS-CoV-2 infection^4,5^.

The persistence of a specific immune response represents a crucial point to be addressed in order to define the risk of re-infection and to optimize the vaccination strategies. In convalescent patients, several data confirmed that both antibodies and T cell response persisted 6–8 months after the infection^5,6^ but no definitive data are available in vaccinated individuals.

In this study, we established the dynamics and persistence of the immune response by assessing the both anti-Region-Binding-Domain (RBD) antibody and Spike (S)-specific T-cell response early and after 12 weeks from vaccination in a cohort of HCWs who received a two-dose BNT162b2-mRNA vaccination at the National Institute for Infectious Diseases L. Spallanzani in Rome.

## Methods

### Enrolled subjects

A convenient sample (n = 78) of HCWs who received BNT162b2 mRNA vaccine were extracted from the cohort of vaccinated HCWs established at the INMI L.Spallanzani. All enrolled HCWs were naïve for SARS-CoV-2 infection as shown by a negative test for anti-Nucleocapside (anti-N) and anti-Spike receptor-binding-domain (anti-RBD) antibodies at T0. Out of 78 HCWs enrolled in the study, 58 (74.3%) were women, median age was 45 years (IQR 26–64). Most enrolled HCWs (n = 59, 76%) had been employed in direct care to COVID-19 patients. The study was approved by the Ethical committees of the National Institute for Infectious Diseases L Spallanzani (issue N. 297/2021) and all HCWs signed an informed consent. All methods were performed in accordance with the relevant guidelines and regulations.

### Antibody evaluation and micro-neutralization assay (MNA_90_)

Two commercial chemiluminescence microparticle antibody assays (ARCHITECT^®^ i2000sr Abbott Diagnostics, Chicago, IL, USA) were used: the anti-Nucleoprotein IgG and the SARS-CoV-2 IgG II kit, detecting antibodies against the RBD of SARS-CoV-2. The neutralization assay was performed according to^7^, using SARS-CoV-2/Human/ITA/PAVIA10734/2020, provided by Fausto Baldanti, Pavia, as challenging virus.

### T-cell immune response

Peripheral blood was collected in heparin tubes and stimulated or not with a pool of peptides spanning the Spike protein (Miltenyi Biotech, Germany) at 37 °C (5% CO_2_), according to^8–10^. A superantigen (SEB) was used as positive control. Plasma were harvested after 16–20 h of stimulation and stored at − 80 °C. Th1-cytokines (IFN-γ, TNF-α, IL-2) were quantified in the plasma samples using an automatic ELISA (ELLA, Protein Simple). The detection limit of these assays were 0.17 pg/ml, 0.3 pg/ml and 0.54 pg/ml for IFN-γ, TNF-α and IL-2 respectively.

### Statistical analysis

Continuous variables including anti-RBD, anti-N, MNA_90_ titers, IFN-γ, TNF-α, IL-2 levels were reported as median and interquartile range (IQR). Comparison of medians across groups were evaluated by Wilcoxon for pairwise comparisons. Correlations between assays were assessed by non-parametric Spearman's rank tests. Analyses were performed using Graph Pad. A 2-sided P value < 0.05 was considered statistically significant.

## Results

**Figure 1:**
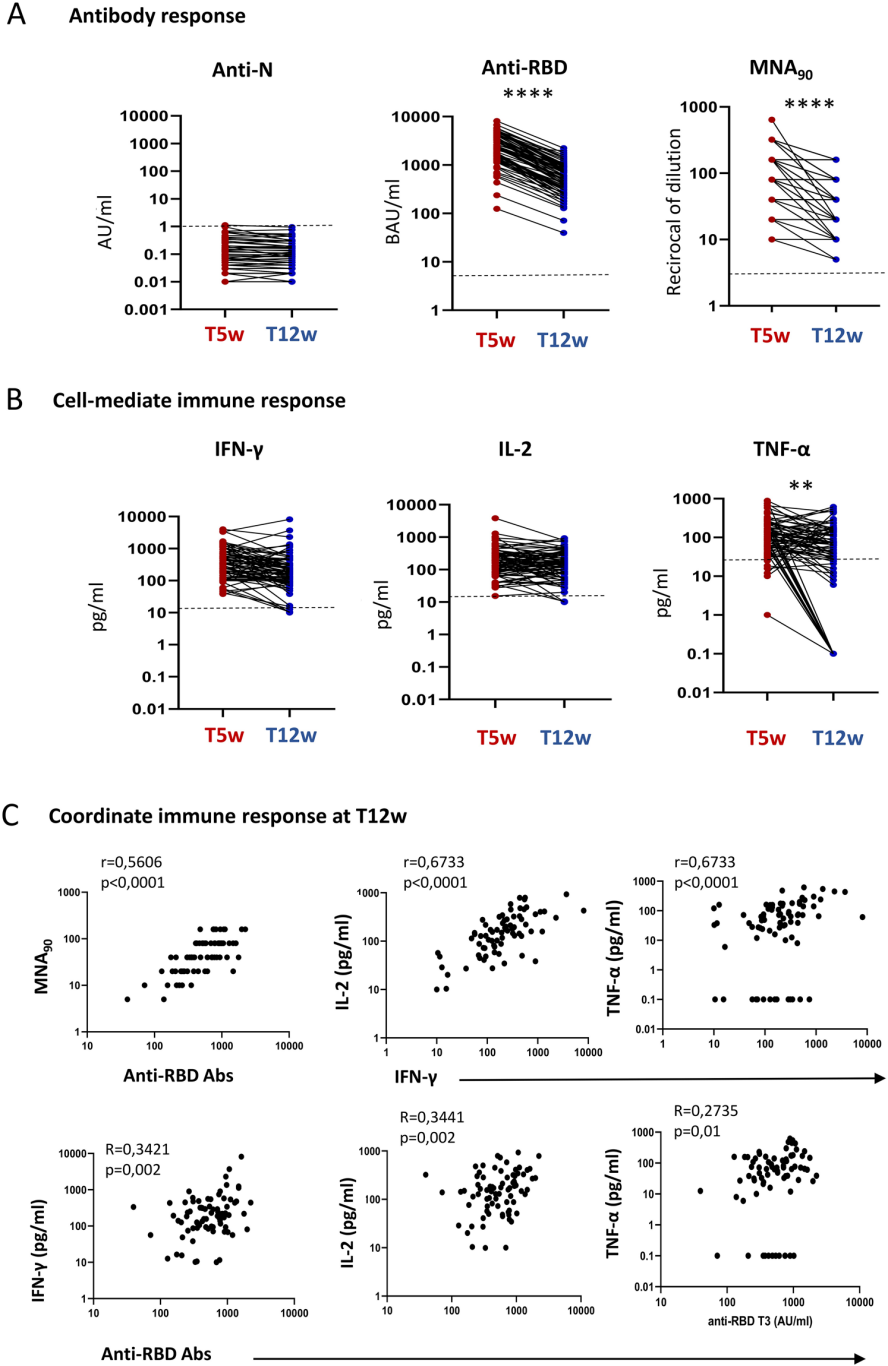
(**A**) Humoral response 5 (T5w) and 12 (T12w) weeks after vaccination. SARS-CoV-2 specific anti-N, anti-RBD and neutralizing Abs were quantified in sera samples at both time points. Anti-N-IgG are expressed as index values S/CO and values ≥ 1.4 are considered positive; Anti-RBD-IgG are expressed as Binding Arbitrary Units (BAU)/mL and values ≥ 7.1 are considered positive; neutralizing Abs are expressed as the highest serum reciprocal of dilution inhibiting at least 90% of the cytopathic effect (MNA_90_), values ≥ 10 are considered positive. (**B**) Cell-mediated immune response 5 (T5w) and 12 (T12w) weeks after vaccination. Cytokines (IFN-γ, TNF-α, IL-2) were quantified in stimulated blood samples at both time points and showed after subtracting the background. Dashed line identify the cut-off of each test calculated as the mean  ± 2SEM of 55 anti-S and anti-N negative HCW before vaccination. Differences between the median in T5w and T12w were calculated by paired t test. ****p < 0.0001; *p < 0.5. (**C**) Correlation between humoral and cell-mediated immune response 12 (T12w) weeks after vaccination. Correlations within humoral levels (anti-RBD and MNA_90_), within cell-mediated response (IFN-γ, TNF-α and IL-2) and across humoral and cell-mediated immunity (anti-RBD and IFN-γ, TNF-α and IL-2) are shown. Correlation was analyzed by Spearmen test. Comparison of medians across groups were evaluated by Wilcoxon test for pairwise comparisons. Correlations between assays were assessed by non-parametric Spearman's rank tests. A 2-sided P value < 0.05 was considered statistically significant.

We assessed the kinetics of humoral- and cell-mediated immune response to BNT162b2-mRNA vaccination in 78 HCWs. Specifically, the enrolled HCWs were sampled 5 (T5w) and 12 (T12w) weeks after vaccination and humoral response was evaluated by anti-RBD antibody, while the natural infection was excluded by anti-N antibody. As shown in Fig. 1, anti-N antibodies were undetectable at both time points, confirming no SARS-CoV-2 natural infection during the study duration (Fig. 1A). In contrast, a detectable anti-RBD antibody response was observed in 100% of HCWs early after vaccination (T5w) as previously published^10^, and persisted in all but one HCWs after 12 weeks from vaccination (T12w). Nevertheless, a significant reduction of anti-RBD titer was observed [median T5w: 2395 BAU/ml (1445–4089) vs. T12w: 569 BAU/ml (308–911), p < 0.0001]. A similar kinetic was observed also when analyzing neutralizing antibody: all HCWs showed a positive neutralization test both at T5w and T12w, but the titer dropped down after 12 weeks from vaccination [median reciprocal of dilution T5w: 80 (80–240) vs. T12w: 40 (20–80), p < 0.0001].

The S-specific T cells was evaluated by quantifying Th1 cytokines (IFN-γ, IL-2 and TNF-α) after stimulation with a pool of peptides covering the S protein (Fig. 1B). All HCW showed a positive specific T cell response and produced IFN-γ (78/78, 100%), IL-2 (78/78, 100%) and TNF-α (72/78, 92.3%). Interestingly, differently from the antibody titer, specific T cells persisted overtime and, although few patients experienced a very slight decrease, the level of IFN-γ [median T5w: 343 pg/ml (IQR: 167.1–703.6) vs. T12w: 199.1 pg/ml (93.7–437.9), p = 0.13], and of IL-2 [median T5w: 177 pg/ml (83.9–305.1) vs. T12w: 156.5 pg/ml (72.3–302.0), p = 0.12] persisted overtime. In contrast, a decrease of TNF-α production was observed (median T5w: 125 pg/ml (60.3–210.3) vs. T12w: 62 pg/ml (12.3–142.8), p < 0.002], probably due to a strong reduction occurred in 15 HCWs. Not particular features of these 15 patients (age, gender, comorbidity) has been reported.

We therefore focused on the immune response at T12w in order to define the coordination of both humoral and cell-mediated immunity (Fig. 1C). A positive correlation between anti-RBD and MNA90 was maintained (r = 0.5606, p < 0.0001), although decrease respect to that observed early after vaccination (r = 0.8103, p < 0.0001). The S-specific T cell response at T12w was characterized by the production by Th1 cytokines and the amount of IFN-γ correlated with IL-2 (r = 0.6733, p < 0.0001) and TNF-α (r = 0.6733, p < 0.0001). Finally, a slight but significant correlation between anti-RBD antibody and IFN-γ (R = 0.3421, p < 0.0001), IL-2 (r = 0.3441, p < 0.0001) and TNF-α (R = 0.2735, p < 0.0001) has been reported, suggesting the persistence of a coordinated humoral and cell-mediated immune response.

## Discussion

The vaccination strategies are aimed to induce a protective immunity with persistent humoral and cell-mediated immune response. In this context, Yellow Fever and smallpox vaccines represent good examples inducing remarkably effective and long-lived immune protection through the induction of neutralizing antibodies and T-cells with broad specificity, high magnitude, polyfunctionality, high proliferative potential and long-term persistence^11^.

In this study, we described the persistence of both humoral and cell-mediated immune response to Spike in HCW after 12 weeks from vaccination. Nevertheless, while anti-RBD and neutralizing titer decreased overtime, the S-specific T cell immunity persisted at similar level.

The ability of BNT162b2 mRNA to induce a strong humoral and cell-mediated immune response early after vaccination was confirmed in several studies^1–3^ but data on their persistence have not still clarified. In convalescent SARS-CoV-2 patients growing evidences support the persistence of the immune response more than 8 months both in symptomatic and in asymptomatic patients^5,12,13^. During the acute phase of infection, the induction of a coordinate immune response, by neutralizing antibodies production and specific T-cell response resulted effective against the virus and represents a main feature of mild disease^4,14^. After recovery, each component of SARSCoV-2 immune memory persisted and differentiate in memory cells^5^.

Here we showed that two dose of BNT162b2 mRNA vaccine in healthy subjects induced a strong anti-RBD and neutralizing antibody response which however begin to decrease after 12 weeks from vaccination prime. The decrease of effector antibody-producing B cells could be associated to the differentiation of the specific memory compartment, as demonstrated after natural infection^5^. Interestingly, despite the antibody reduction, a persistent T cell immunity has been observed and S-specific T lymphocytes produced IFN-γ and IL-2 at similar extent as early after vaccination, suggesting the maintenance of a robust pool of effector Ag specific T cells until 12 weeks. The ability to respond to specific stimulation producing several Th1 cytokines may represent a good marker of protective phenotype as shown in convalescent COVID-19 patients^13^. In particular, the production of IL-2 can be essential for the homeostatic maintenance of a functional specific T-cell response. Moreover, we found a positive correlation between the two arms of the immune system that can be important to generate a balanced memory pool of B and T lymphocytes. A reduced level of neutralizing antibody at 6 months post-vaccination as well as memory B and T cells was reported^15^. Of note, vaccine-induced memory B cells can recognize the variants of concern showing a high hypermutation in variant-binding B cell clones, suggesting a continued evolution to fight new variants.

Overall, our study describes the different kinetic of anti-RBD and Cell-mediated immune response after 12 weeks from BNT162b2 mRNA vaccine in a cohort of HCWs. Despite a significant decrease of antibody, the specific T cells able to produce several Th1 cytokines persisted as early after booster dose to elicit a coordinated neutralizing humoral and cytokine producing S-specific T-cell response in HCW. The study has been conducted in a single center and recruited healthy, young and middle aged adults, predominantly female, without previous SARS-CoV-2 infection, and its findings should not be generalized to different population.

Assessing the dynamics of these parameters is essential to evaluate the long-term protection and to establish its duration. These results will be crucial to generate the evidence needed for a better understanding of the optimal vaccination strategy.

## Data Availability

The data presented in this study are available on request from the corresponding author.
